# Efficacy of Mitomycin C in Endoscopic Dacryocystorhinostomy: A Systematic Review and Meta-Analysis

**DOI:** 10.1371/journal.pone.0062737

**Published:** 2013-05-13

**Authors:** Shi-ming Cheng, Yi-fan Feng, Ling Xu, Yan Li, Jin-hai Huang

**Affiliations:** 1 Department of Ophthalmology, Taihe Hospital Affiliated to Hubei University of Medicine, Hubei, China; 2 Department of Ophthalmology, Zhongshan Hospital, Fudan University, Shanghai, China; 3 Department of Ophthalmology, Dongfeng Hospital Affiliated to Hubei University of Medicine, Hubei, China; 4 The Affiliated Eye Hospital of Wenzhou Medical College, Zhejiang, China; Medical University Graz, Austria

## Abstract

**Background:**

A number of published comparative studies have been conducted to evaluate the efficacy and safety of intraoperative mitomycin C (MMC) in endoscopic dacryocystorhinostomy (EN-DCR). However, results have not always been consistent. Therefore, we carried out a meta-analysis to compare the clinical results of EN-DCR with and without MMC.

**Methods and Findings:**

A comprehensive literature search of Cochrane Library, PubMed and EMBASE to identify relevant trials comparing EN-DCR with and without MMC. Eleven studies including 574 eyes were included in this meta-analysis. The success was defined as patency of the nasolacrimal canal and symptomatic improvement. There was significantly higher success rate in the MMC group in comparison with control group [RR = 1.12, 95% CI (1.04, 1.20), P = 0.004]. A sensitivity analysis after the non-randomized controlled trials were excluded from the meta-analysis demonstrated no differences compared with the overall results. Subgroup analyses showed that MMC group had a significantly higher success rate than control group in primary and revision EN-DCR, and EN-DCR without silicone intubation, but no difference in the subgroup of with silicone intubation. The size of the osteotomy site was bigger in the MMC group compared to the control group at 3 months [WMD = 7.65, 95% CI (0.33, 14.98), P = 0.041] and 6 months [WMD = 9.28, 95% CI (2.45, 16.11), P = 0.008] after surgery. However, there was statistically significant difference in the osteotomy surface area between the two groups at 12 months after surgery [WMD = 11.63, 95% CI (−1.04, 24.29), P = 0.072].

**Conclusion:**

Intraoperative MMC application seems to be a safe adjuvant that could reduce the closure rate of the osteotomy and enhance the success rate after both primary and revision EN-DCR.

**Trial Registration:**

ClinicalTrials.gov NCT01772277

## Introduction

Nasolacrimal duct obstruction (NLDO) is a common cause for ophthalmologic evaluation. The advent of dacryocystorhinostomy (DCR) provided a revolution in the management of tearing secondary to nasolacrimal duct obstruction. It can be performed through an external or endonasal approach. The endonasal approach was introduced by Caldwell in 1893 [1], and modernized by the endonasal endoscopic technique in 1989 proffered by McDonough and Meiring [2]. Endoscopic dacryocystorhinostomy (EN-DCR) has been accepted as a highly successful procedure, because of less surgical trauma, shorter operative and hospitalization times, low postoperative discomfort, and greater cosmetic accessibility [3]. In a recent systematic review of outcomes after DCR in adults, the success rate of EN-DCR was found to range from 84 to 94% [4].

Mitomycin C (MMC) is an antineoplastic agent that inhibits the synthesis of DNA, cellular RNA, and protein by inhibiting the synthesis of collagen by fibroblasts [5]. MMC was originally used as a systemic chemotherapeutic agent, it has been widely used in ophthalmic practice both intraoperatively and postoperatively for prevention of pterygium recurrence, enhancing the success rate of glaucoma filtration surgery [6]–[8]. Recently, use of MMC has been described in lacrimal drainage surgery. A previous meta-analysis conducted by us found that intraoperative MMC application was a safe adjuvant that could reduce the closure rate of the osteotomy site after primary external dacryocystorhinostomy (EX-DCR) [9].

Recently, many controlled trials have investigated adjunctive MMC for primary or revision EN-DCR to augment the surgical success rate, but the results are not completely consistent [10]–[15]. Some studies [10]–[12] have found that the application of MMC improved the success rate of EN-DCR, whereas others [13]–[15] suggested the use of intraoperative MMC in EN-DCR surgery did not change the success rate of this procedure. To the best of our knowledge, there was no meta-analysis on comparison of success rate of EN-DCR with MMC (MMC group) and EN-DCR without MMC (control group). Therefore, the aim of this study was to undertake systematic review and meta-analysis to evaluate the efficacy of intraoperative MMC application in EN-DCR surgery and help ophthalmologists to determine whether it is a useful adjuvant in EN-DCR surgery.

## Materials and Methods

Following generally accepted methodology recommendations, this meta-analysis was performed according to the PRISMA (Preferred Reporting Items for Systematic Review and Meta-Analyses) statement (Text S1) [16]. The investigators wrote a protocol and registered it with the ClinicalTrials.gov Protocol Registration System (identification number: NCT01772277) in January 2013 [17].

### Search Strategy

Reports of clinical trials comparing EN-DCR with and without MMC were identified through a systematic search. The following electronic databases were searched: PubMed, Embase and the Cochrane Central Register of Controlled Trials from January 1, 1990 to December 30, 2012. A comprehensive search was conducted using the following terms “dacryocystorhinostomy”, “mitomycin C” and “nasolacrimal duct obstruction”. Language restrictions were not used. Retrieved studies from both PubMed and Embase were imported into Refworks (version 1.0; Refworks, Bethesda, MD) where duplicate articles were manually deleted. Titles and abstracts of the remaining studies were independently scanned by 2 authors (S.M.C. and Y.F.F.). The full texts of the potentially relevant reports were then read to determine whether they met our inclusion criteria. In addition, the reference lists from all identified studies were also examined.

### Inclusion Criteria

The following selection criteria were used to identify published studies for inclusion in this meta-analysis: (1) comparative studies; (2) adult patients (>18 years) with NLDO undergoing primary or revision EN-DCR; and (3) all studies included were required to provide the success rates of both MMC and control groups, and the followed up time was more than 6 months. The following were excluded: (1) studies which did not provide the success rates; and (2) studies which included pediatric cases. Where multiple publications based on the same cohort were identified, the report with the largest number of patients was used.

### Data Extraction

Date extraction and quality assessment was performed according to the customized protocol by two reviewers (S.M.C. and Y.F.F.) independently. We extracted the following data from the eligible studies: (1) general characteristics (title, first author, journal and year of publication); (2) methodology (type of study, country of origin, sequence generation, allocation concealment, masking or blinding, incomplete outcome data, selective reporting and other sources of bias); (3) subjects (recruitment site, enrollment periods, inclusion criteria, exclusion criteria, general patient characteristics); (4) Interventions (concentration of MMC and expose time); (5) types of EN-DCR (primary and recurrent); (6) outcomes (measurement, follow-up time and loss of follow-up); (7) analysis (statistical methods); (8) results (quantitative results and qualitative results). Any disagreement was resolved by discussion or consensus involving a third reviewer (J.H.H.) when necessary.

### Quality Assessment

The qualities of randomized controlled trials (RCTs) were assessed by two independent observers (S.M.C. and Y.F.F.) using a Jadad composite scale [18], allocating 1 point for the presence of each of the following: randomization, masking and participant withdrawals/dropouts. If randomization and blinding were appropriate, 1 additional point was added for each. Thus, the total score ranged from 0 to 5. Studies scoring less than 3 points were considered to be of low quality. In addition, the Newcastle-Ottawa Scale (NOS) [19] was used to evaluate only non-RCTs and the selection, comparability and outcome or exposure for cohort or case-control studies. The maximum for selection was 4 *, for comparability was 2 * and for outcome or exposure was 3 *. The maximum NOS score was 9 *, and the studies with ≥6 * were considered to have relatively higher quality.

### Outcome Measures

The primary outcome measure was success rate, which was determined by the presence of any one of the following: (1) patent lacrimal passage on syringing, (2) symptomatic improvement, and (3) endoscopic visualisation of fluorescein dye at the nasal opening of the anastomoses. The secondary outcome measure that we have reviewed was the difference in ostium size between patients operated with EN-DCR with MMC and patients operated with EN-DCR without MMC. We also reviewed the most common adverse events that were reported such as haemorrhage, infection, granulation or wound dehiscence.

### Statistical Analysis

The meta-analysis was conducted using Stata software package (version 11.0; Stata Corp., College Station, TX). The success rate was treated as dichotomous variables, whereas ostium size was treated as continuous variable. Dichotomous data were presented as relative ratio (RR) with 95% confidence interval (CI). Weighted mean differences (WMD) with 95% CI were calculated for continuous variables. Both ORs and WMDs were considered statistically significant at the P<0.05 level.

Statistical heterogeneity was analyzed using a chisquare test [20]. The *I^2^* statistic was calculated to assess heterogeneity between studies (P<0.10 was considered representative of significant statistical heterogeneity). If there was heterogeneity between studies, a random-effects model was carried out using the DerSimonian- Laird method; otherwise, a fixed effects model was used for pooling the data. Sensitivity analysis was performed to examine the effect of excluding non-randomized studies and subgroup analysis was performed according types of EN-DCR (primary EN-DCR and revision EN-DCR) and whether silicone tubes were used. Potential publication bias was estimated by both visually evaluating a funnel plot and the Egger test [21], [22].

## Results

### Study Selection

**Figure 1:**
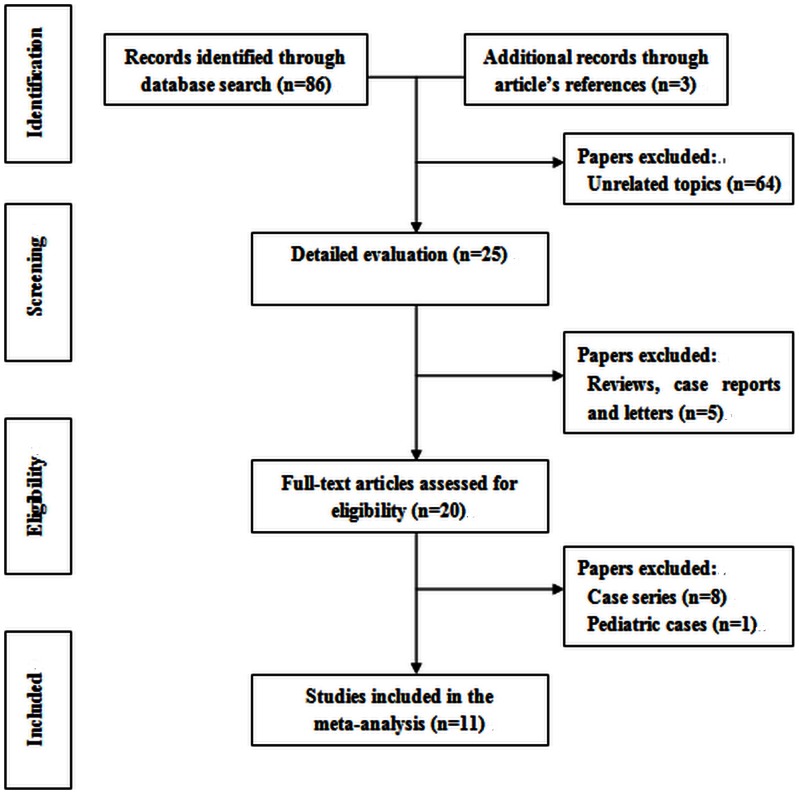
Search and study selection process.

The selection of studies is summarized in Figure 1. A total of 89 articles were initially identified; 86 records were identified in the database search, and 3 records were found in article reference lists. However, only 19 of these studies investigated the effect of MMC on EN-DCR surgery in adult. Of these 19 articles, 8 were noncomparative case series [23]–[30] and not suitable for inclusion in the meta-analysis (Table 1). The left 11 comparative studies (including 9 RCTs and 2 non-RCTs) which met our inclusion criteria were included in the final meta-analysis [10]–[15], [31]–[35].

**Table 1:** Summary of the characteristics of excluded non-comparative studies.

*First Author/Year*	*Location*	*No. Eyes*	*Silicone* *Tube Use*	*Success* *Rate (%)*	*Mean Follow-Up (range, mo)*	*MMC* *Concentration*	*MMC Expose Time*
Selig [23]/2000	USA	8	Yes	88	10	0.4 mg/mL	3 to 5 min
Zilelioğlu [24]/2002	Turkey	64	Yes	80	11.3 (6–60)	0.5 mg/mL	2.5 min
Yuen [25]/2004	China	99	Yes	80	(17.6–25.6)	0.4 mg/mL	5 min
Nemet [26]/2007	Australia	5	Yes	80	15.4 (8–19)	0.3 mg/mL	unknown
Dolmetsch [27]/2010	Colombia	224	Yes	95	18.2 (6–108)	0.5 mg/mL	10 min
Apuhan [28]/2011	Turkey	22	Yes	91	18 (6–30)	0.5 mg/mL	2.5 min
Görgülü [29]/2012	Turkey	20	Yes	90	17 (12–24)	0.2 mg/mL	5 min
Mak [30]/2012	China	83	Yes	94	23.3	0.2 mg/mL	3 to 10 min

### Characteristics of Eligible Studies

The studies were published between 1998 and 2012, and comprised a total of 574 eyes (291 in the MMC group and 283 in the control group). One study was divided into two comparative groups as it included both primary and revision DCRs. Three studies were done in India [12], [13], [33], 3 in Turkey [11], [30], [32], and 1 each in Iran [15], Finland [34], Egpty [35], Thailand [14], and China [10]. The mean age of patients in most of studies ranged from 32 to 70 years and the percentage of female patients ranged from 42% to 90%. Sample sizes in these studies ranged from 30 to 92. The mean follow-up period ranged from 6 to 18.2 months. The dose of MMC used ranged between 0.2 and 0.5 mg/mL, which was placed on the ostium for 2 to 15 minutes. Silicone tubes were used in five studies [11], [14], [15], [31], [35]. Table 2 presents the characteristics of the included studies.

**Table 2:** Summary of the characteristics of included studies.

*First Author/Year*	*Location*	*Design*	*MMC* *Concentration*	*MMC Expose* *Time*	*No. Eyes (MMC/control)*	*Gender (male: female)*	*Follow-Up* *(mo)*	*Silicone Tube Use*	*Types of DCR*
Qin [10]/2010	China	RCT	0.4 mg/mL	3 min	73 (39/34)	12∶23/9∶21	12	Yes	Primary
Ghosh [33]/2006	India	RCT	0.2 mg/mL	2 min	30 (15/15)	12∶18	12	No	Primary
Prasannaraj [13]/2012	India	RCT	0.2 mg/mL	10 min	38 (17/21)	16∶22	6	No	Primary
Mudhol [12]/2012	India	RCT	0.2 mg/mL	5 min	60 (30/30)	15∶45	12	No	Primary
Tirakunwichcha [14]/2011	Thailand	RCT	0.5 mg/mL	3 min	50 (26/24)	4∶22/5∶19	12	Yes	Primary
Farahani [15]/2008	Iran	RCT	0.2 mg/mL	3 and 15 min	92 (46/46)	11∶35/13∶33	12.17±1.18/12.80±1.52	Yes	Primary
Penttilä [34]/2011	Finland	RCT	0.4 mg/mL	5 min	30 (15/15)	2∶13/1∶14	6	No	Recvison
Özkiriş [11]/2012	Turkey	RCT	0.5 mg/mL	5 min	36 (18/18)	11∶7/10∶8	11.5/12.7	Yes	Recvison
Ragab [35]/2012	Egpty	RCT	0.5 mg/mL	10 min	76 (38/38)	27∶49	12	Yes	Recvison
Zilelioğlu [31]/1998	Turkey	Non-RCT	0.5 mg/mL;	2.5 min	40 (22/18)	13∶26	18.2	Yes	Primary and Recvison
Özkiriş [32]/2012	Turkey	Non-RCT	0.5 mg/mL	5 min	54 (28/26)	12∶16/12∶14	14.3/13.2	No	Primary

RCT = randomized-controlled trials; DCR = dacryocystorhinostomy; MMC = Mitomycin C.

### Quality Assessment

The quality assessment of RCTs is shown in Table 3. In 5 of all the RCTs included in the systematic review, the investigators described a random component in the sequence generation process such as: referring to a random number table [13] or using a computer random number generator [11], [34] or using a random blocks [14], [35]. The remainder did not describe the specific methods of random sequence generation. Of 5 RCTs that described their masking or binding, 4 used double-blinding [11], [14], [15], [35] and 1 used single-blinding [13]. All RCTs described the data of missing patients and only one study had missing cases: 6 of 76 (6.6%) [35]. In addition, the scores of NOS scale for the remainder two non-RCTs were 5 [31] and 6 [32] respectively.

**Table 3:** Summary of the methodological quality of included randomized controlled trials.

*First Author/Year*	*Jadad Score*					
	Randomization	Appropriateness of Randomization	Single/Double Blind	Appropriateness of Blind	Withdrawals	*Sum of Jadad Score*
Qin [10]/2010	1	0	0	0	1	2
Ghosh [32]/2006	1	0	0	0	1	2
Prasannaraj [13]/2012	1	1	1	1	1	5
Mudhol [12]/2012	1	0	0	0	1	2
Tirakunwichcha [14]/2011	1	1	1	1	1	5
Farahani [15]/2008	1	0	1	1	1	4
Penttilä [33]/2011	1	1	0	0	1	3
Özkiriş [11]/2012	1	1	1	1	1	5
Ragab [34]/2012	1	1	1	1	1	5

### Success Rates

**Figure 2:**
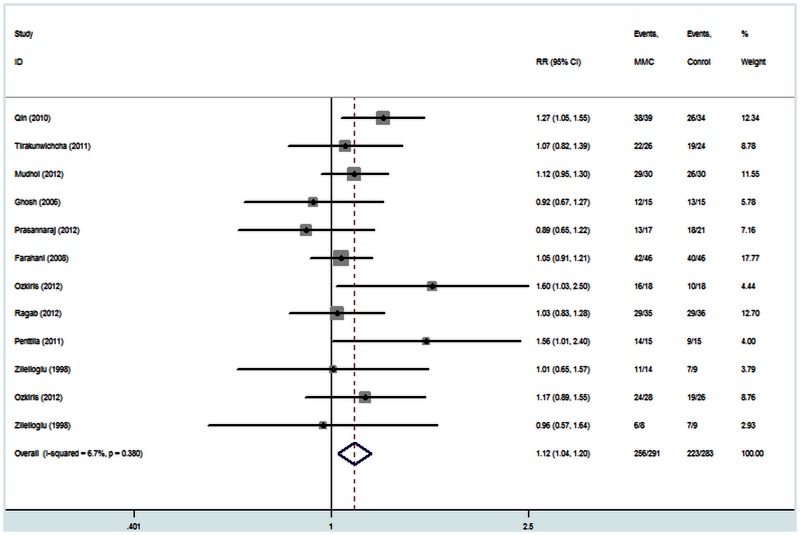
The success rate of endoscopic dacryocystorhinostomy with and without Mitomycin C.

**Figure 3:**
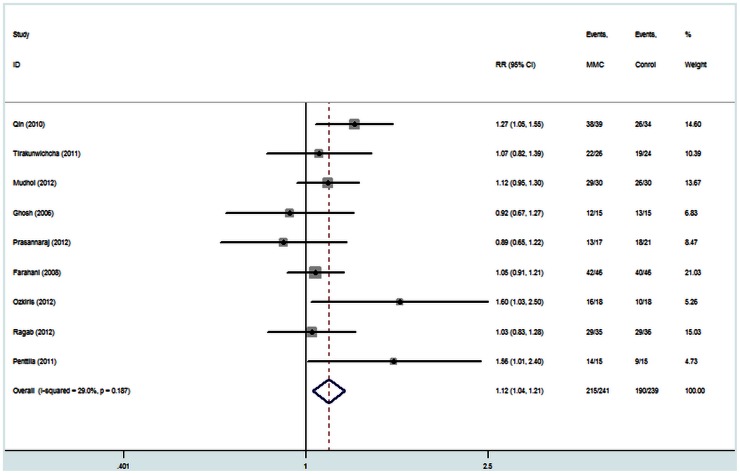
Sensitivity analysis of the success rate of endoscopic dacryocystorhinostomy with and without Mitomycin C.

All of the eleven studies reported data for success rates. There was no statistical evidence of heterogeneity across these studies (I^2^ = 6.7%, P = 0.38), so the fix effect model was used for meta-analysis. The results are shown in Figure 2. Examination of the forest plot revealed that success rates of the MMC group were significantly higher than those of the control group [RR = 1.12, 95% CI (1.04, 1.20), P = 0.004]. A sensitivity analysis was performed to examine the effect of excluding the nonrandomized studies [31], [32], but this did not alter the above results [RR = 1.12, 95% CI (1.04, 1.21), P = 0.005; Figure 3].

### Subgroup Analysis

**Figure 4:**
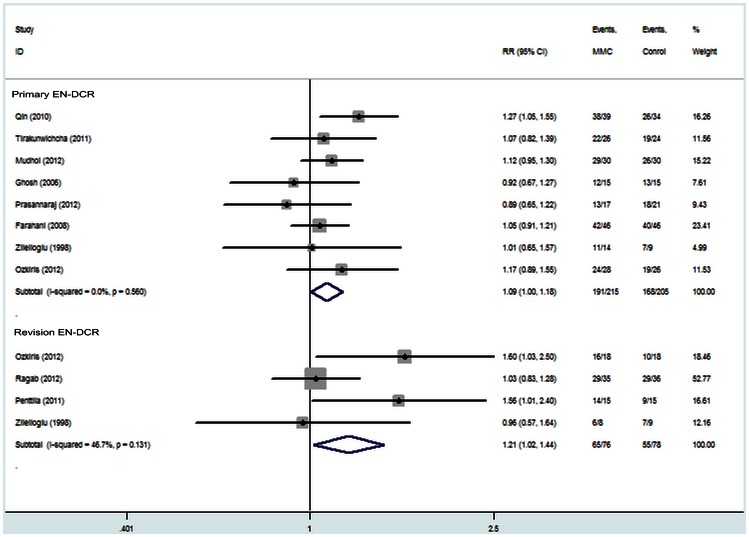
Subgroup analysis of the success rate of endoscopic dacryocystorhinostomy with and without Mitomycin C.

**Figure 5:**
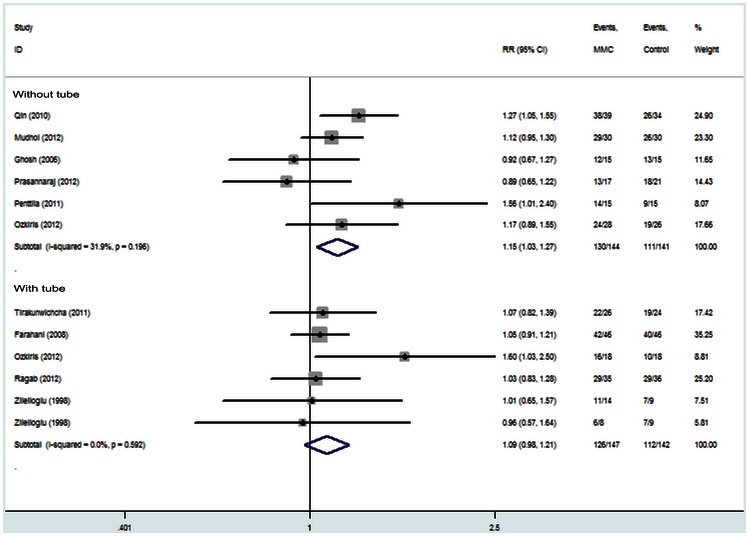
Subgroup analysis of the success rate of endoscopic dacryocystorhinostomy with and without Mitomycin C.

The four subgroups were used to produce the fix effect model for success rates. The results are as follows: Subgroup 1 (primary EN-DCR, eight studies [10], [12]–[15], [31]–[33], recruiting 420 eyes) [RR = 1.09, 95% CI (1.00, 1.18), P = 0.045; Figure 4]; Subgroup 2 (revision EN-DCR, four studies [11], [31], [34], [35], recruiting 144 eyes) [RR = 1.21, 95% CI (1.02, 1.45), P = 0.029; Figure 4]; Subgroup 3 (EN-DCR without silicone intubation, six studies [10], [12], [13], [32]–[34], recruiting 285 eyes) [RR = 1.15, 95% CI (1.03, 1.27), P = 0.010; Figure 5]; Subgroup 4 (EN-DCR with silicone intubation, five studies [11], [14], [15], [31], [35], recruiting 289 eyes) [RR = 1.09, 95% CI (0.98, 1.21), P = 0.122; Figure 5]. All the results indicated that MMC group had a higher success rate except the subgroup of EN-DCR with silicone intubation.

### Ostium Size

**Figure 6:**
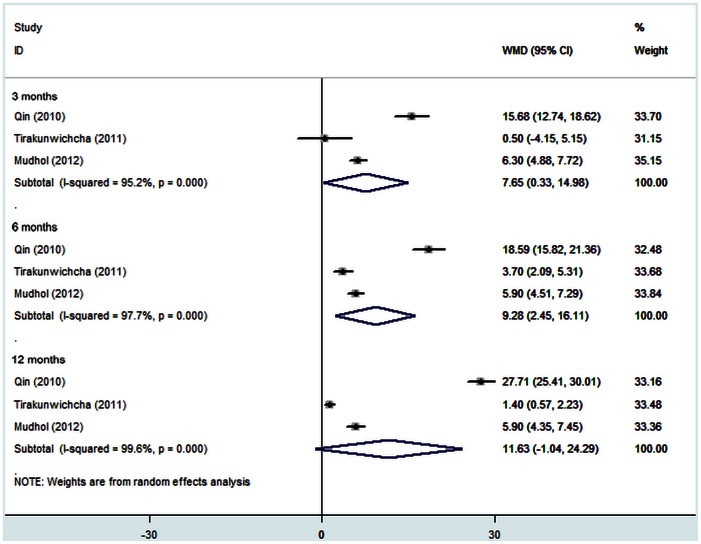
The mean ostium size of endoscopic dacryocystorhinostomy with and without Mitomycin C after surgery.

Three studies (recruiting 183 eyes) [10], [12], [14] compared the mean size of the osteotomy site at 3 months, 6 months and 12 months after surgery. The results are shown in Figure 6. All three subgroups had heterogeneity of effect size (P>0.10), so the fixed effect model was used for meta-analysis. Examination of the forest plot revealed that the size of the osteotomy site was bigger in the MMC group compared to the control group at 3 months [WMD = 7.65, 95% CI (0.33, 14.98), P = 0.041] and 6 months [WMD = 9.28, 95% CI (2.45, 16.11), P = 0.008] after surgery. However, there was no significant difference in the osteotomy surface area between the two groups at 12 months after surgery [WMD = 11.63, 95% CI (−1.04, 24.29), P = 0.072].

### Adverse Events

No MMC-related complications were reported in all studies. Total seven cases of synechia were recorded in two studies [13], [35]: three cases in the MMC group and four cases in the control group. Moreover, Prasannaraj and associates [13] found granulations were seen in the stomal margins of two successful patients in the MMC group and six successful patients in the control group.

### Publication Bias

**Figure 7:**
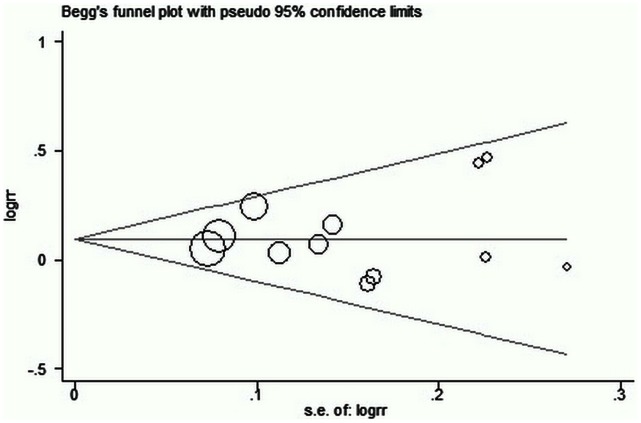
Begg’s funnel plot for the success rate of endoscopic dacryocystorhinostomy.

Publication bias is the term for what occurs when the research that appears in the published literature is systematically unrepresentative of the population of completed studies: The strongest and most positive studies are most likely to be published. An assessment using the Begg rank correction (Figure 7) and the Egger test (P = 0.73) demonstrated no evidence of publication bias.

## Discussion

Findings from the present meta-analysis indicate that adjunctive intraoperative MMC application with EN-DCR surgery had a significantly higher success rate than EN-DCR surgery without MMC. Sensitivity and subgroup analysis also suggested that the results were comparatively reliable. Moreover, in three studies included in this meta-analysis, mean ostium size was significantly bigger in MMC group than that in control group at 3, 6 months postoperatively. However, the difference of mean ostium size was not significant between two groups at 12 months postoperatively.

The most common reason for the failure of this operation is the formation of scar or granulation tissue over the rhinostomy site [36], [37] It is postulated that adjunctive use of MMC over the osteotomy site in EN-DCR surgery could inhibit scarring and granulation tissue formation around the osteotomy site or common canaliculus and enhance the success of EN-DCR surgery. Based on our meta-analyses, it appears that it will be helpful to apply MMC over the osteotomy site to increase the success rate of primary EN-DCR (P = 0.045). For revision DCR, the endoscopic approach is especially superior to the external approach. The normal scarring produced after the external incision makes a revision procedure very uncomfortable, and the final aesthetic and functional results are usually poor. In contrast, endoscopic revision DCR is an easy procedure with mainly good results [38], [39]. Moreover, Korkut and associates [40] evaluated the results of primary and revision EN-DCR. They stated that EN-DCR is a safe and effective procedure in revision cases, as well as in primary cases. In the present meta-analysis, only four studies of revision EN-DCR were included. Although the results showed that the success rate was higher in the MMC group than control group (P = 0.029), future larger sample size comparative clinical trials are needed to prove it.

Only three trials compared the ostium size between MMC groups and control group after surgery [10], [12], [14]. All these studies clearly showed the mean size of the ostium 3, 6 and 12 months postoperatively was bigger in the MMC group than in the control group, though the difference was not significant at 12 months [WMD = 11.63, 95% CI (−1.04, 24.29), P = 0.072]. This manifestation strongly supports the antifibrotic property of the MMC in maintaining the ostium size in the postoperative period. In addition, Mudhol and associates [12] shown that there is a small reduction in the size of the lacrimal ostium in the first 4 weeks which corresponds to the initial stages of healing. However, after 4 weeks there was no significant change in ostium size. Their findings correlate with the results of Mann and associates [41]. Furthermore, the large reduction in sizes might reflect variations in surgical techniques and the strong healing process and remodeling that could be different in ethnicity [14].

There is a difference of opinion as to whether a silicone tube should be inserted. To prevent obliteration of the intranasal lacrimal sac ostium, many surgeons prefer to insert either bi- or monocanalicular silicone tubes to stent the internal ostium. However, it has been postulated that silicone tubing itself may cause tissue granulation, predisposing the site to postoperative infection and adhesions, and canalicular lacerations, resulting in surgical failure [42], [43]. Thus, some surgeons suggested the use of MMC to suppress fibrous proliferation and scar formation during EX-DCR surgery with silicone intubation. The results of the subgroup analysis demonstrated that there was no significant difference between patients undergoing silicone intubation accompanied by MMC application and silicone intubation alone during EX-DCR (P = 0.122).

Some complications such as corneal ulcus, corneal perforations, scleral calcification, secondary cataract, endophthalmitis, hypotony and maculopathy have been reported from the use of MMC in pterygium and glaucoma surgery [44], [45]. Nevertheless, the application of MMC in EN-DCR appears to be safe, and the occurrence of complications was at a relatively low level. No articles included in this study reported MMC-related complications such as abnormal nasal bleeding, mucosal necrosis, or infection.

After surgery, there is a natural tendency for the stoma to contract during the healing process, hence, the follow-up period must be adequate to accommodate completion of this healing process. An analysis by Boush and associates [46] showed that most surgical failures occurred within the first 4 months after surgery. Similar findings were reported by Kong and associates [47] who observed that the average onset of stomal closure after primary operation was 12.7 weeks. Woog and associates [48] reported that the average onset of failure was 7.5 weeks postoperatively. Therefore, only studies with a minimum follow-up period of 6 months were selected in this systematic review. However, most patients included in this meta-analysis were relatively young. A recently retrospective study [49] reported that in EN-DCR for primary acquired nasolacrimal duct obstruction, younger patient age at time of surgery was associated with a higher rate of failure. However, we did not perform an analysis of this subgroup of patients with different ages due to lack of data.

This meta-analysis may have some limitations. First, two comparative studies included in the analysis were not randomized, which may leave them vulnerable to bias. Although the Begg and the Egger test demonstrated no evidence of publication bias, the results should be interpreted with caution publication bias. Second, a potential source of heterogeneity in the results was the different concentrations and exposure time of MMC application. In the current systematic review of eleven studies, the dose of MMC used ranged between 0.2 and 0.5 mg/mL and the exposure time from 2–15 minutes. Thus, a further controlled study with a large sample size is needed to evaluate the optimum concentration, as well as applied duration of MMC for EN-DCR. This may provide more conclusive information for determining whether intraoperative MMC in EN-DCR is a safe and effective adjuvant.

In summary, this meta-analysis suggests that intraoperative MMC application seems to be a safe adjuvant that could help achieve favorable success rates and reduce the closure rate of the osteotomy site after EN-DCR.

## Supporting Information

Text S1 PRISMA checklist.
(PDF)

## References

[1] Caldwell GW (1893) Two new operations for obstruction of the nasal duct with preservation of the canaliculi. Am J Ophthalmol 10: 189–191.

[2] McDonogh M, Meiring JH (1989) Endoscopic transnasal dacryocystorhinostomy. J Laryngol Otol 103: 585–587. 2769026 10.1017/s0022215100109405

[3] Korkut AY, Teker AM, Yazici MZ, Kahya V, Gedikli O, et al (2010) Surgical outcomes of primary and revision endoscopic dacryocystorhinostomy. J Craniofac Surg 21: 1706–1708. 21119404 10.1097/SCS.0b013e3181f3c6c1

[4] Leong SC, Macewen CJ, White PS (2010) A systematic review of outcomes after dacryocystorhinostomy in adults. Am J Rhinol Allergy 24: 81–90. 20109333 10.2500/ajra.2010.24.3393

[5] Wakaki S, Marumo H, Tomioka K (1958) Isolation of new fractions of antitumor mitomycins. Antibiot Chemother 8: 228–240. 24544727

[6] Singh G, Wilson MR, Foster CS (1988) Mitomycin eye drops as treatment for pterygium. Ophthalmology 95: 813–821. 3211484 10.1016/s0161-6420(88)33104-0

[7] Mégevand GS, Salmon JF, Scholtz RP, Murray AD (1995) The effect of reducing the exposure time of mitomycin C in glaucoma filtering surgery. Ophthalmology 102: 84–90. 7831047 10.1016/s0161-6420(95)31049-4

[8] Palmer SS (1991) Mitomycin as adjunct chemotherapy with trabeculectomy. Ophthalmology 98: 317–321. 2023752 10.1016/s0161-6420(91)32293-0

[9] Feng YF, Yu JG, Shi JL, Huang JH, Sun YL, et al (2012) A meta-analysis of primary external dacryocystorhinostomy with and without mitomycin C. Ophthalmic Epidemiol. 19: 364–370. 10.3109/09286586.2012.733792 23171205

[10] Qin ZY, Lu ZM, Liang ZJ (2010) Application of mitomycin C in nasal endoscopic dacryocystorhinostomy. Int J Opbthalmol (Guoji Yanke Zazhi) 10: 1569–1571.

[11] Özkiriş M, Ozkiriş A, Göktaş S (2012) Effect of mitomycin C on revision endoscopic dacryocystorhinostomy. J Craniofac Surg 23: e608–10. 23172494 10.1097/SCS.0b013e31826c7cf7

[12] Mudhol RR, Zingade ND, Mudhol RS, Das A (2012) Endoscopic Ostium Assessment Following Endonasal Dacryocystorhinostomy with Mitomycin C Application. Al Ameen J Med Sci 5: 320–324.

[13] Prasannaraj T, Kumar BY, Narasimhan I, Shivaprakash KV (2012) Significance of adjunctive mitomycin C in endoscopic dacryocystorhinostomy. Am J Otolaryngol 33: 47–50. 21392851 10.1016/j.amjoto.2011.01.001

[14] Tirakunwichcha S, Aeumjaturapat S, Sinprajakphon S (2011) Efficacy of mitomycin C in endonasal endoscopic dacryocystorhinostomy. Laryngoscope 121: 433–436. 21271601 10.1002/lary.21292

[15] Farahani F, Ramezani A (2008) Effect of intraoperative mitomycin C application on recurrence of endoscopic dacryocystorhinostomy. Saudi Med J 29: 1354–1356. 18813431

[16] Liberati A, Altman DG, Tetzlaff J, Mulrow C, Gotzsche PC, et al (2009) The PRISMA statement for reporting systematic reviews and meta-analyses of studies that evaluate health care interventions: explanation and elaboration. PLoS Med 6: e1000100 doi:10.1371/journal.pmed.1000100. 19621070 10.1371/journal.pmed.1000100 PMC2707010

[17] Feng YF (2011) Efficacy of Mitomycin C in Endoscopic Dacryocystorhinostomy: A Systematic Review and Meta-Analysis. ClinicalTrials.gov. Available: http://clinicaltrials.gov/ct2/show/NCT01772277. Accessed 2013 Jan 18. 10.1371/journal.pone.0062737 PMC3652813 23675423

[18] Jadad AR, Moore RA, Carroll D, Jenkinson C, Reynolds DJ, et al (1996) Assessing the quality of reports of randomized clinical trials: is blinding necessary? Control Clin Trials 17: 1–12. 8721797 10.1016/0197-2456(95)00134-4

[19] Stang A (2010) Critical evaluation of the Newcastle-Ottawa scale for the assessment of the quality of nonrandomized studies in meta-analyses. Eur J Epidemiol 25: 603–605. 20652370 10.1007/s10654-010-9491-z

[20] Higgins JP, Thompson SG (2002) Quantifying heterogeneity in a meta-analysis. Stat Med 21: 1539–1558. 12111919 10.1002/sim.1186

[21] Begg CB, Mazumdar M (1994) Operating characteristics of a rank correlation test for publication bias. Biometrics 50: 1088–1101. 7786990

[22] Egger M, Davey Smith G, Schneider M, Minder C (1997) Bias in meta-analysis detected by a simple, graphical test. BMJ 315: 629–634. 9310563 10.1136/bmj.315.7109.629 PMC2127453

[23] Selig YK, Biesman BS, Rebeiz EE (2000) Topical application of mitomycin-C in endoscopic dacryocystorhinostomy. Am J Rhinol 14: 205–207. 10887629 10.2500/105065800782102672

[24] Zílelíoğlu G, Tekeli O, Uğurba SH, Akiner M, Aktürk T, et al (2002) Results of endoscopic endonasal non-laser dacryocystorhinostomy. Doc Ophthalmol 105: 57–62. 12152803 10.1023/a:1015702902769

[25] Yuen KS, Lam LY, Tse MW, Chan DD, Wong BW, et al (2004) Modified endoscopic dacryocystorhinostomy with posterior lacrimal sac flap for nasolacrimal duct obstruction. Hong Kong Med J 10: 394–400. 15591598

[26] Nemet AY, Wilcsek G, Francis IC (2007) Endoscopic dacryocystorhinostomy with adjunctive mitomycin C for canalicular obstruction. Orbit 26: 97–100. 17613855 10.1080/01676830601174627

[27] Dolmetsch AM (2010) Nonlaser endoscopic endonasal dacryocystorhinostomy with adjunctive mitomycin C in nasolacrimal duct obstruction in adults. Ophthalmology 117: 1037–1040. 20079535 10.1016/j.ophtha.2009.09.028

[28] Apuhan T, Yıldırım YS, Eroglu F, Sipahier A (2011) Effect of mitomycin C on endoscopic dacryocystorhinostomy. J Craniofac Surg 22: 2057–2059. 22067858 10.1097/SCS.0b013e3182319863

[29] Görgülü O, Ozdemir S, Görgülü FF, Altin A, Selçuk T, et al (2012) Adjunctive use of mitomycin C in endoscopic revision dacryocystorhinostomy. B-ENT 8: 123–126. 22896931

[30] Mak ST, Io IY, Wong AC (2012) Prognostic factors for outcome of endoscopic dacryocystorhinostomy in patients with primary acquired nasolacrimal duct obstruction. Graefes Arch Clin Exp Ophthalmol (In press). 10.1007/s00417-012-2228-9 23250482

[31] Zilelioğlu G, Uğurbaçs SH, Anadolu Y, Akiner M, Aktürk T (1998) Adjunctive use of mitomycin C on endoscopic lacrimal surgery. Br J Ophthalmol 82: 63–66. 9536884 10.1136/bjo.82.1.63 PMC1722355

[32] Özkiris M, Özkiris A (2012) Endoscopic dacryocystorhinostomy not using canalicular silicone intubation tube with and without mitomycin C: a comparative study. Eur J Ophthalmol 22: 320–325. 21948027 10.5301/ejo.5000048

[33] Ghosh S, Roychoudhury A, Roychaudhuri BK (2006) Use of mitomycin C in endo-DCR. Indian J Otolaryngol Head Neck Surg 58: 368–369. 23120350 10.1007/BF03049597 PMC3450354

[34] Penttilä E, Smirnov G, Seppä J, Kaarniranta K, Tuomilehto H (2011) Mitomycin C in revision endoscopic dacryocystorhinostomy: a prospective randomized study. Am J Rhinol Allergy 25: 425–428. 22185749 10.2500/ajra.2011.25.3676

[35] Ragab SM, Elsherif HS, Shehata EM, Younes A, Gamea AM (2012) Mitomycin C-enhanced revision endoscopic dacryocystorhinostomy: a prospective randomized controlled trial. Otolaryngol Head Neck Surg 147: 937–942. 22645114 10.1177/0194599812450280

[36] Jokinen K, Kärjä J (1974) Endonasal dacryocystorhinostomy. Arch Otolaryngol 100: 41–44. 4842611 10.1001/archotol.1974.00780040045009

[37] Allen KM, Berlin AJ, Levine HL (1988) Intranasal endoscopic analysis of dacrocystorhinostomy failure. Ophthal Plast Reconstr Surg 4: 143–145. 10.1097/00002341-198804030-00004 3154733

[38] Tsirbas A, Davis G, Wormald PJ (2005) Revision dacryocystorhinostomy: a comparison of endoscopic and external techniques. Am J Rhinol 19: 322–325. 16011142

[39] Korkut AY, Teker AM, Ozsutcu M, Askiner O, Gedikli O (2011) A comparison of endonasal with external dacryocystorhinostomy in revision cases. Eur Arch Otorhinolaryngol 268: 377–381. 20652292 10.1007/s00405-010-1339-3

[40] Korkut AY, Teker AM, Yazici MZ, Kahya V, Gedikli O, et al. 2010 Surgical outcomes of primary and revision endoscopic dacryocystorhinostomy. J Craniofac Surg 21: 1706–1708. 21119404 10.1097/SCS.0b013e3181f3c6c1

[41] Mann BS, Wormald PJ (2006) Endoscopic Assessment of the dacryocystorhinostomy ostium after endoscopic surgery. Laryngoscope 116: 1172–1174. 16826055 10.1097/01.mlg.0000218099.33523.19

[42] Woog JJ, Kennedy RH, Custer PL, Kaltreider SA, Meyer DR, et al (2001) Endonasal dacryocystorhinostomy: a report by the American Academy of Ophthalmology Ophthalmology. 108: 2369–2377. 10.1016/s0161-6420(01)00945-9 11733286

[43] Unlu HH, Toprak B, Aslan A, Guler C (2002) Comparison of surgical outcomes in primary endoscopic dacryocystorhinostomy with and without silicone intubation. Ann Otol Rhinol Laryngol 111: 704–709. 12184592 10.1177/000348940211100809

[44] Rubinfield RS, Pfister RR, Stein RM, Foster CS, Martin NF, et al (1992) Serious complications of topical mitomycin C after pterygium surgery. Ophthalmology 99: 1647–1654. 1454338 10.1016/s0161-6420(92)31749-x

[45] Zacharria PT, Depperrman SR, Schuman JS (1993) Ocular hypotony after trabeculectomy with mitomycin C. Am J Ophthalmol. 116: 314–336. 10.1016/s0002-9394(14)71349-2 8357056

[46] Boush GA, Lemke BN, Dortzbach RK (1994) Results of endonasal laser-assisted dacryocystorhinostomy. Ophthalmology 101: 955–959. 8190487 10.1016/s0161-6420(94)31231-0

[47] Kong YT, Kim TI, Kong BW (1994) A report of 131 cases of endoscopic laser lacrimal surgery. Ophthalmology 101: 1793–1800. 7800358 10.1016/s0161-6420(94)31100-6

[48] Woog JJ, Metson R, Puliafito CA (1993) Holmium: YAG endonasal laser dacryocystorhinostomy. Am J Ophthalmol 116: 1–10. 8328525 10.1016/s0002-9394(14)71736-2

[49] Mak ST, Io IY, Wong AC (2012) Prognostic factors for outcome of endoscopic dacryocystorhinostomy in patients with primary acquired nasolacrimal duct obstruction. Graefes Arch Clin Exp Ophthalmol. In press. 10.1007/s00417-012-2228-9 23250482

